# Settler-colonialism, social acceptance, and support for reparations in Canada

**DOI:** 10.1177/02697580251338312

**Published:** 2025-07-16

**Authors:** Andreea Ioana Zota, Ismehen Melouka, Jo-Anne Wemmers

**Affiliations:** Université de Montréal, Canada; Université de Montréal, Canada; Université de Montréal, Canada

**Keywords:** Collective victimization, needs-based model of reconciliation, reparations, settler-colonialism, social acceptance

## Abstract

In contexts of collective victimization such as settler colonialism in Canada, recognizing both historical and ongoing victimization, as well as supporting reparations measures, is crucial for healing the relationship between Indigenous and non-Indigenous peoples and for propelling reconciliation efforts forward. While most non-Indigenous Canadians recognize the historical victimization of Indigenous peoples, fewer acknowledge ongoing victimization of Indigenous communities and support measures promoting their right to self-determination. The recognition of ongoing inequality can evoke feelings of guilt and pose a threat to the privileges of the advantaged groups, which may lead to minimizing and disregarding experiences of victimization of the disadvantaged group. While recognition of ongoing victimization can be uncomfortable, it is not impossible. According to the needs-based model, members of the advantaged group may be more willing to acknowledge the ongoing structural victimization and consider relinquishing their privileges if they receive social acceptance from members of the disadvantaged group. Using data (*n* = 313) collected from an original survey, this paper examines if social acceptance by Indigenous peoples influences: (a) acknowledgment of ongoing victimization, (b) support for justice measures, and (c) recognition of Indigenous autonomy among non-Indigenous Canadians. The findings suggest that there is a positive relationship between social acceptance and both recognition of victimization and support for justice measures. However, no significant association was found between social acceptance and support for autonomy. These findings partially support the needs-based model, suggesting that social acceptance plays a limited role in promoting reconciliation.

## Introduction

The colonization of North America by Europeans meant the violent destruction of its Indigenous Peoples. For generations, settler colonialism operated with the aim of establishing a settler population through land dispossession and the erasure of Indigenous presence (Veracini, 2010; Wolfe, 2006). In Canada, Indian Residential Schools (IRS) were introduced in the 19th century to force the assimilation of Indigenous Peoples into European culture^1^. While colonialism has been in decline since the Second World War, it was not until 1996 that the last Indian Residential School in Canada finally closed its doors. Due to its structural nature (Wolfe, 2006), settler colonialism continues to have profound socioeconomic consequences for Indigenous Peoples. Historical colonial policies, such as the IRS system, have left a legacy of intergenerational trauma, chronic health issues, and economic disadvantages (Bombay et al., 2013, 2020; Kaspar, 2014; Kim, 2019; Smallwood et al., 2021).

Globally for countries dealing with legacies of mass atrocities and human rights violations, such as colonization, transitional justice has emerged as a dominant framework (Gready and Robins, 2014; Lambourne, 2009). This approach is founded on four fundamental pillars: truth-seeking, aimed at uncovering the past; accountability of perpetrators, involving the recognition of victimization and acknowledgment of the harm-doers’ responsibility; reparations, which encompass various measures addressing the consequences of harm; and institutional reforms to prevent future harm (Elster, 2004; Quinn, 2009). Ending victimization necessitates its recognition. Non-Indigenous Canadians play a crucial role in challenging and disrupting the settler mindset (Regan, 2010). In the context of colonization, this involves recognizing the atrocities committed against Indigenous people, acknowledging the State’s responsibility for their suffering, and taking measures to repair the consequences of colonialism (Quinn, 2009; Wemmers, 2014).

Despite ongoing disparities, non-Indigenous Canadians, in particularly those who have lived in Canada for several generations referred to as Settlers (Regan, 2010), often fail to fully recognize the continuous nature of Indigenous victimization and are hesitant to support measures that promote Indigenous self-determination (Canadian Reconciliation Barometer, 2022; Environics Institute Survey Research, 2019). If victimization is not recognized, it is unlikely to stop, which poses a serious challenge for any efforts to promote reconciliation and healing. This paper explores how reconciliation in Canada might be facilitated. After a brief examination of the historical context of settler-colonialism in Canada and the socioeconomic disparities between Indigenous and non-Indigenous populations, reconciliation efforts are discussed. The importance of recognition and its implications are examined, followed by an explanation of the needs-based model. After a description of the methodology, the data analyses are presented. This paper concludes with a discussion of the findings and a conclusion.

This paper contributes to reconciliation studies by examining the role of *social acceptance* in enhancing non-Indigenous Canadians’ recognition of Indigenous victimization and support for justice measures. Using a *quantitative survey methodology* with university students from Saskatchewan and Quebec, the study finds that while social acceptance fosters acknowledgment of past and ongoing harm, it has limited influence on support for Indigenous autonomy, highlighting the challenges of achieving deeper reconciliation and transformative justice.

## Recognition of collective victimization

Recognition of victimization and acknowledgment of the suffering experienced by victims are crucial for their healing, well-being, and empowerment (Twali et al., 2020; Vollhardt, 2020; Wemmers, 2017; Wemmers and Manirabona, 2014). Acknowledging these systemic inequalities can evoke feelings of guilt and threaten the privileges of advantaged groups (Jost and Banaji, 1994; Tajfel, 1981), leading to resistance against recognizing Indigenous rights and supporting reparative actions. People are generally motivated to maintain a positive image of their social group (Lerner, 1980; Tajfel, 1981). Admitting that their in-group has committed atrocities poses a challenge for reconciliation, but without it change is unlikely.

Failing to acknowledge their victimhood can lead to revictimization and hinder the healing process (Ferrari et al., 2023; Wemmers, 2017). Acknowledgment is a multifaceted concept that extends beyond mere recognition of one’s victimization. Twali et al. (2020) identify four levels of acknowledgment, grouping various interpretations and definitions of recognition found in social psychology, peacebuilding and reconciliation literature. They note that in this line of research it is often implied that recognizing a group’s collective victimization is essential for healing, yet this concept is rarely treated as a distinct construct (Twali et al., 2020: 297). While several reconciliation models address recognition (see, for instance, Vollhardt et al., 2022), Twali et al.’s framework is particularly relevant to our study in defining recognition of collective victimization and applying it to the Canadian context.

The first level is factual acknowledgment of collective victimization, which includes recognizing *who* was targeted and *what* happened (Twali et al., 2020). This would entail recognizing negative consequences of colonial policies, like the IRS policy and its legacy for victims. For some scholars, it includes the historical and ongoing experiences of displacement, assimilation, and violence endured by Indigenous Peoples despite constitute genocide (MMIWG, 2019; TRC, 2015; Woolford and Benvenuto, 2015). Most Canadians do recognize the historical victimization of Indigenous Canadians, however, few are willing to label it as genocide (Woolford and Benvenuto, 2015). Three in five Canadians say they are very (17%) or somewhat (43%) familiar with the history of Indian residential schools in Canada (Environics Institute Survey Research, 2021: 17).

The second level goes beyond acknowledgment of past events and delves into the recognition of the ongoing suffering experienced by the victimized group. Termed ‘Empathic Acknowledgment’ by Twali et al. (2020), this level entails acknowledging the psychological, social, and economic ramifications resulting from the harm, as demonstrated above. This first and second level can be understood as what Quinn describes as ‘thin sympathy’ in the Canadian context, which involves a basic awareness and understanding of historical and ongoing events (Quinn, 2016). Although many Canadians acknowledge the historical impacts of colonization, they are less likely to recognize the ongoing effects of colonial policies. (Canadian Reconciliation Barometer, 2022; Environics Institute Survey Research, 2019). What is clear, however, is that support for addressing the injustices resulting from settler colonialism is moderate (Canadian Reconciliation Barometer, 2022; Environics Institute Survey Research, 2019). This holds particularly true for Canadians whose families have lived in Canada for several generations, who often exhibit more negative attitudes toward reconciliation and Indigenous Peoples compared to recent immigrants (Beauvais, 2022; Melouka, 2019). Putting an end to the ongoing cycle of victimization requires fully recognizing the consequences of colonialism and supporting measures of reparation (Wemmers, 2014).

The third level of acknowledgment involves the perpetrator’s acceptance of responsibility for the harm inflicted. Victim groups often demand that members of the perpetrator group acknowledge their wrongdoing (Iqbal and Bilali, 2018; Twali et al., 2020). This entails a public acknowledgment by the perpetrator group that their actions violated fundamental moral principles and supporting reparations measures (Iqbal and Bilali, 2018). It involves acknowledging their contribution to the other’s victimization. While recognition of historic victimization is high among Canadians, it remains unclear whether they recognize that ongoing socioeconomic inequalities are the result of past harm and extent to which they recognize their own responsibility in perpetuating these inequalities.

The fourth level takes the acknowledgment process a step further with the ‘Negative Identity Internalization by Perpetrators’. In this stage, wrongdoers are required to accept the negative aspect of their identity (Shnabel et al., 2021; Twali et al., 2020). It goes beyond acknowledging the wrongdoing committed by their group; they must also embrace it as a negative component of their social identity (Shnabel et al., 2021). This deeper level of acknowledgment is crucial, as victims often express dissatisfaction with shallow apologies or reconciliation. It is one of the most challenging forms of acknowledgment, as it requires deconstructing prevailing narratives of the peaceful Canadian, and tarnishing the image of Canada as a nation known for its politeness and peacefulness (Henry et al., 2010; Regan, 2010).

Further, the process of internalizing a negative collective self-conception is complex and may not occur in a linear fashion. Scholarship in settler colonial studies offers theoretical insights into the challenges of moving from surface-level acknowledgment to deeper internalization. Ermine’s (2007) work on the concept of ethical space emphasizes its role in bridging Indigenous and non-Indigenous worldviews, creating a neutral space for respectful and reciprocal dialogue, while Hiller’s (2016) concept of spirals of unsettlement suggests that while acknowledgment of injustices is often met with resistance, it can also represent cumulative shifts in settler consciousness, pushing white settlers into action in solidarity with Indigenous peoples or leading others to reflect inwardly on uncomfortable truths about their relationship to the land, which may reinforce support for the status quo. Similarly, Davis et al. (2017) explore the nonlinear and contradictory pathways individuals take when grappling with historical injustice, while Bacon (2017) examines the emotional and psychological barriers that impede deeper acknowledgment. These frameworks suggest that internalizing negative identity components may be shaped by ongoing contestations over national identity, land dispossession, and settler privilege, which complicate efforts toward genuine reconciliation.

In Canada, we appear to be at an early stage of acknowledgment, recognizing the past victimization of Indigenous Peoples, but not necessarily ongoing victimization. While progress has been made and recognition of the harm caused by the IRS policy has increased in recent years (Canadian Reconciliation Barometer, 2022), the prevalence of denialism is also increasing, and misinformation remains a significant concern (Taylor, 2023). Denialism undermines recognition and poses a challenge for reconciliation.

## The enduring impacts of colonialism in Canada

Over the years, there have been numerous reports and other efforts to recognize the negative consequences of colonization for Indigenous people in Canada. Perhaps the most significant of these is the public apology for the residential school system issued by the Prime Minister of Canada in 2008, in which he acknowledged the harm inflicted by IRS on Indigenous children and the lasting negative consequences on Indigenous language, culture, and heritage. Recognizing its responsibility for the suffering by the former IRS students, the federal government established the Indian Residential School Settlement Agreement, providing financial compensation to survivors of these institutions.

That same year, the federal government funded the Truth and Reconciliation Commission (TRC). The TRC’s landmark report in 2015, which included 94 Calls to Action across various sectors such as education, health, youth, justice, and language, ultimately seeks to foster reconciliation between Indigenous and non-Indigenous communities (TRC, 2015)^2^. One year later, after years of hesitation and active resistance, Canada announced its full support for the United Nations Declaration on the Rights of Indigenous Peoples (UNDRIP). In 2021, the UNDRIP received Royal Assent and became formally recognized in Canadian law, thereby affirming the rights of Indigenous Peoples (Government of Canada, Department of Justice, 2021).

Despite the end of the IRS system, Indigenous people continue to face severe socioeconomic disadvantages as a result of historical colonial policies (Bombay et al., 2020; Hajizadeh et al., 2018; Kaspar, 2014; Lamb and Verma, 2021; Pendakur and Pendakur, 2011). There are still approximately 40,000 residential school survivors today (de Bruin, 2020), Survivors of the IRS system and their families experience intergenerational trauma, chronic health problems, low incomes, high unemployment rates, and low levels of education (Bombay et al., 2013, 2020; Kaspar, 2014; Kim, 2019; Smallwood et al., 2021; Wilson and Macdonald, 2010). The children of survivors, who themselves never experienced residential school, exhibit signs of post-traumatic stress (Bombay et al., 2013). Hence, colonial violence extends beyond the boundaries of residential schools, and settler-colonialism in Canada persists to this day.

This reality becomes particularly evident when examining national statistics on key socioeconomic indicators. National data reveal a widening gap between Indigenous and non-Indigenous populations in multiple areas, including victimization, employment, education, housing, health outcomes, and incarceration rates (Bleakney et al.,2020; Cotter, 2022; Government of Canada, 2022; Pendakur and Pendakur, 2011; Perrault, 2022; Posca, 2018). The consistently higher rate of victimization among Indigenous Peoples and especially women and girls eventually led to a National Inquiry into Missing and Murdered Indigenous Women and Girls (MMIWG). Using a legal framework, the Inquiry found that Canada’s past and current colonial policies toward Indigenous Peoples constitute genocide. It called on governments to end the ongoing genocide of Indigenous people in Canada and address root causes of violence (MMIWG, 2019). These deeply embedded inequalities within structures, systems, and institutions emphasize the long-term consequences of colonialism and underscore the imperative to take action in addressing them (Chartrand, 2019; Milward, 2022).

Despite the many efforts to recognize the systemic victimization of Indigenous Peoples in Canada, minimal progress has been observed (Breen et al., 2022). The recognition of ongoing inequalities has become a highly political issue and has not been fully embraced across the country. Deeply rooted in the social, economic, and political systems that shape and govern Canadian society, changing them requires commitment from all levels of government. In 2020, Canada’s Prime Minister recognized the presence of structural and systemic inequalities, which involves the unequal distribution of resources, opportunities, and power within Canadian society^3^. However, acknowledgment is not shared equally across Canada’s provinces and territories (Letourneau, 2024). Overall, eastern Canada, which was colonized first, tends to have more favorable attitudes toward Indigenous-non-Indigenous relations than the Prairie provinces in western Canada, such as Saskatchewan (Environics Institute Survey Research, 2024). Non-Indigenous Canadians residing in the Prairie provinces tend to hold more ambivalent views and show less overall sympathy toward the challenges faced by Indigenous Peoples than non-Indigenous Canadians in the east (Beauvais, 2022; Environics Institute Survey Research, 2019; Letourneau, 2024). Quebeckers consistently exhibit more favorable views regarding the recognition of unique Indigenous Peoples’ rights than the Prairies (Environics Institute Survey Research, 2016, 2019).

## Moving toward reconciliation

Drawing from principles of transitional justice (Elster, 2004; Galtung, 2011; Quinn, 2009), *reconciliation* processes represent a profound journey wherein conflicting parties strive to build trust and forge lasting peace (Lerche, 2000). It is important to keep in mind that multiple frameworks of reconciliation exist, and through our research, we identified patterns that are useful in understanding how reconciliation can be effectively pursued in Canada, particularly by addressing key factors that facilitate or hinder this process. Reconciliation has become a goal for many in Canada (TRC, 2015) and is widely recognized as pivotal in addressing colonial victimization, confronting historical and structural injustices (Bashir, 2012), offering avenues for reparations and healing for victims (Wemmers, 2014), and fostering trust and equity between Indigenous and non-Indigenous populations (Regan, 2010).

However, these processes are difficult and not linear, as they require acknowledgment of ongoing injustices (Regan, 2010), collective accountability for past harm (Neufeld et al., 2022), and support for reparations (Nagy, 2022; UN, 2007). In Canada, they should challenge or ‘unsettle’ established national narratives by recognizing the realities of how colonial politics played and are still playing a role in eradicating Indigenous identities, cultures, and social bonds (McGuire and Denis, 2019; Nagy, 2022; Regan, 2010; Wotherspoon and Milne, 2021). They should aim to redefine the relationships and dynamics between Indigenous and non-Indigenous peoples. While unsettling and challenging on both an individual and collective level, these processes demand from all Canadians recognition of ongoing violence, accountability and deconstruction of national narratives.

Despite its grassroots origins, the TRC, as a truth-seeking mechanism of transitional justice, faced criticism for its soft approach to reconciliation and the lack of structural actions following the release of its final report (Denis and Bailey, 2016; Landry, 2020; Lincez, 2022). Many of the reports previously stated can also be viewed as representing a soft perspective on reconciliation, emphasizing emotional aspects of restoring harmony while making limited progress in implementing concrete measures to enhance the lives of Indigenous Peoples. For instance, settlements, such as those provided to survivors of the IRS, are often seen as a way to move forward. However, they frequently fall short in adequately addressing the needs of the victims (Landry, 2020; Petoukhov, 2022; Wolfe, 2006). While compensation may be offered, it often serves as a temporary solution, leaving survivors with unmet basic needs and even contributing to family conflicts (Willick, 2018). The formal apology issued by the Prime Minister of Canada, though perceived by many as a symbolic gesture, lacks substantive actions to bring about meaningful change, which impedes reconciliation.

The attitudes of non-Indigenous Canadians mirror this soft perspective of reconciliation. Past surveys indicate they often emphasize the importance of fostering harmonious relationships between Indigenous and non-Indigenous peoples and moving beyond historical grievances (Canadian Reconciliation Barometer, 2022; Environics Institute Survey Research, 2019; Wotherspoon and Milne, 2021). This perspective raises several concerns as it fails to address the underlying causes of structural and systemic violence and to recognize the rights and needs of Indigenous Peoples (Corntassel, 2012; Short, 2005). For many Canadians, the TRC and other national and provincial inquiries may mean truth was uncovered, justice was served, and that it is now time to move on from the past.

Recognizing the limitations of transitional justice, recent advancements in the literature highlight a shift toward transformative justice mechanisms. Transformative justice focuses on grassroots initiatives to restore justice and address ongoing inequalities through a bottom-up approach, emphasizing the necessity of change across the entire population. In contrast, truth commissions and other transitional justice mechanisms tend to mainly focus on specific events and individuals, whereas transformative justice addresses broader structural and systemic injustices.

It is a victim-centered process that tackles underlying structural violence, prioritizing the rights and needs of victims, and aims for transformative, enduring change and lasting peace (Balasco, 2018; Daly, 2002[2001]; Hamber and Lundy, 2020; Mani, 2005; Nagy, 2022). When reconciliation is viewed through the lens of transformative justice, it is regarded as a process that must confront collective victimization and uphold the rights and needs of Indigenous peoples (Nagy, 2022). This perspective advocates for a grassroots approach, placing accountability on non-Indigenous Canadians and striving to rectify systemic injustices. Accountability starts with recognition of past and ongoing harm; therefore, it must be prioritized as a crucial step before engaging in the reconciliation process. As Landry (2020) stresses, failing to do so keeps ‘reconciliation for the colonizer’. Simply put, the current state of reconciliation falls short of being an Indigenous-centered process that fosters transformative change. Reconciliation cannot be a transformative process if it is centered only on the needs of the non-Indigenous Canadians, serving as a means to alleviate guilt or evade responsibility for past actions.

Establishing nation-to-nation relationships and breaking the cycle of ongoing in- equalities involves recognizing the rights of Indigenous Peoples as human rights, as established in the United Nations Declaration on the Rights of Indigenous Peoples (UN, 2007)^4^. Self-determination is central to Indigenous Peoples’ rights and must be a prerequisite for reconciliation (Cornell, 2015; Corntassel, 2012; Palmater, 2015; UN, 2007). Self-determination addresses the fundamental needs of victims who have experienced violence, aiming to restore autonomy and self-efficacy. Acknowledging the nationhood and governance of Indigenous Peoples is crucial for fostering sustainable relationships and abolishing imbalanced power dynamics between Canadians and Indigenous Peoples (Barker, 2015; Corntassel, 2012; Gobby et al., 2022; Palmater, 2015). This includes supporting reparations and Indigenous resistance, such as blockades and land defense efforts, as legitimate forms of governance (Barker and Battell Lowman, 2016; Gobby et al., 2022). Topics related to land rights are politically divisive (Canadian Reconciliation Barometer, 2022; Environics Institute Survey Research, 2019), with recent immigrants and minority groups generally showing high levels of support, while Canadians whose families have lived in Canada for several generations, referred to as ‘Settlers’ by Regan (2010), are less inclined to support reparation measures and recognize Indigenous unique rights (Beauvais, 2022; Melouka, 2019; Starzyk et al., 2019).

Recognizing inequalities and Indigenous self-determination are crucial for reconciliation, but receive less recognition from these ‘old’ Canadians, despite being essential steps toward justice, reparations, and reconciliation. If reconciliation is to be achieved, it is important to understand barriers to recognition and identify factors that could facilitate recognition. For this we turn to social psychology, and in particular, the needs-based model of reconciliation (Shnabel and Nadler, 2008).

## The needs-based model of reconciliation

Social psychologists have been striving to understand why individuals are hesitant to acknowledge victims. Social identity theory (Lerner, 1980; Tajfel, 1981) can shed light on this phenomenon. According to social identity theory, individuals are generally motivated to maintain a positive image of their social group. Admitting that their in-group has committed atrocities tarnishes the moral reputation of the group (Tajfel, 1981). To preserve the belief that their group is good and deserving of respect, group members tend to reject anything that threatens this positive image. Our natural inclination toward our in-group often leads to the presence of explicit and implicit biases and prejudices (Dovidio and Gaertner, 2010; Tajfel, 1981).

For instance, Canadians may justify structural and systemic inequality to preserve their privileges and maintain a positive self-image, leading to feelings of guilt and privilege threat for advantaged groups, which can result in employing system-legitimizing ideologies and holding prejudiced views of disadvantaged groups (Jost, 2019; Jost and Banaji, 1994; Jost and van der Toorn, 2012). White identity in Canada has been linked to Indigenous resentment, implicit and explicit racism, and lack of support for economic redistribution (Beauvais and Stolle, 2022; Harell et al., 2014). The fear of being judged by society or disadvantaged groups, known as image shame, drives a defensive need to restore moral reputation without changing moral conduct (Nooitgedagt et al., 2021; Shnabel et al., 2021). When accused of benefiting from an unfair system that disadvantages marginalized groups, individuals may become defensive and resistant to social change (Hässler et al., 2019; Jost, 2019). Maintaining a positive social identity is a significant factor impeding or facilitating recognition, particularly among Canadians with direct connections to European settlers.

The needs-based model of reconciliation posits that meeting the needs of individuals within a group enhances their willingness to engage in reconciliation efforts (Shnabel and Nadler, 2008; Shnabel et al., 2009, 2021). For instance, when representatives of a victim group express moral and social acceptance toward the perpetrator group, thereby restoring their moral identity, it is expected to foster greater readiness among perpetrator group members to reconcile with the victim group compared to positive messages that are unrelated to the moral-social dimension (Shnabel et al., 2021). When the positive social image of the perpetrator group is threatened, members of that group are more likely to strongly justify the wrongdoings committed by their own group (Hässler et al., 2021; Jost and Hunyady, 2003; Jost et al., 2008; Leach et al., 2007). Shnabel and her colleagues (2008, 2009) found that members of the advantaged group were less likely to recognize the victimization and the autonomy of the disadvantaged group when they did not feel accepted. However, when their moral value was reassured by members of the disadvantaged group, they held more positive attitudes toward the disadvantaged group and showed more willingness to reconcile. Previous studies on interracial interactions have demonstrated that participants’ concerns about being seen as prejudiced, which involve their negative anticipations regarding how others perceive them, can adversely impact these interactions (Dovidio et al., 2002; Livingstone et al., 2020). Acceptance or perceived understanding by outgroup members can enhance trust and intergroup relations (Livingstone et al., 2020). Studies on reconciliation using the Needs-based model indicate that this level of acknowledgment is easier when a message of acceptance was conveyed by the disadvantaged groups highlighting the morality of the advantaged group^5^. A sense of understanding and acceptance within the group fosters positive social dynamics. Recognizing harm may be facilitated by social acceptance, providing members of the advantaged group with a mechanism to manage the negative emotions constructively and avoid stigmatization (Shnabel and Nadler, 2008; Shnabel et al., 2009).

## Importance of the study

While socioeconomic disadvantages faced by Indigenous communities are well documented, there is limited knowledge regarding the role of social acceptance on enhancing recognition of these inequalities and their support for addressing them among non-Indigenous Canadians. This paper aims to address two key questions: First, does social acceptance from Indigenous groups enhance the recognition of historical and ongoing victimization among non-Indigenous Canadians? Second, does social acceptance contribute to increased support for self-determination of Indigenous Peoples and justice reforms aimed at rectifying historical injustices?

## Methodology

The data for this study were collected as part of a larger survey (*n* = 313) examining non-Indigenous Canadians’ attitudes toward reconciliation. The study received ethics approval from the Université de Montréal (CERSC-2019-122-D), which adheres to the Tri-Council Policy Statement (see https://ethics.gc.ca/eng/policy-politique_tcps2-eptc2_2018.html, 2018). Concretely, the survey explores attitudes of non-Indigenous Canadians whose families have resided in Canada for more than three generations (aka *Settlers*) in the provinces of Saskatchewan and Quebec. Understanding the attitudes of these individuals is crucial as they have historically held significant power in all aspects of society at the national level and play a pivotal role in the reconciliation process. They may also demonstrate resistance when it comes to giving up their privileges and thus engaging in reconciliation and be less quick to recognize ongoing victimization of Indigenous peoples in Canada than new immigrants to Canada. In addition, these Canadians may have familial connections with European settlers, making them more inclined to need social acceptance from Indigenous Peoples (Denis, 2020; Shnabel et al., 2021).

The choice of Saskatchewan, a Prairie province, and Quebec, which is in eastern Canada, provides a diverse range of viewpoints. It is particularly relevant considering the presence of multiple Indigenous communities in both provinces. Respondents were recruited through a campus-wide email sent to University of Montreal and University of Regina students of all academic levels and Facebook pages belonging to student groups at these two universities, in June 2020. In order to participate in the survey, respondents had to be third-generation, Canadian, university students of all academic levels, aged 18 years or older. Respondents were compensated $20 each for their participation in the survey. Survey participation was anonymous. Upon completion of the survey, participants who opted for compensation were redirected to a separate page to submit their email, which was later deleted to preserve anonymity.

It is important to note that the sample of participants (*n* = 313) is not representative of all third-generation Canadians. For instance, there is a higher proportion of women compared to men in the sample, which is common in student surveys (Smith, 2008). Furthermore, students are in a transitional phase of life, where both their identity and material circumstances are less settled compared to later stages, making them more prone to change. They may be more liberal, more open to influence, and have fewer material stakes in issues related to Indigenous victimization and redress. This context is important to consider, as it may heighten the impact of social approval and reduce variability in their recognition of injustice and support for remedial measures. While this may be a limitation, the convenience of the sample and the significance of their ongoing and future roles in the reconciliation process remain key factors.

Table 1 presents an overview of the sample’s socio-demographic characteristics. It is important to note that education was not included in the survey as all of the respondents were university students. The average age of the respondents is 25 years old, with the youngest being 18 and the oldest being 56 years old (SD = 6.2 years, median = 23 years old).

**Table 1.** Descriptive statistics.

	N	Mean	SD	Min	Max
Age	313	24.80	6.23	18.00	56.00
Gender	311	0.22	0.41	0.00	1.00
Income (in $CAD)	278	65,692.45	31,605.53	25,000.00	100,000
Province	293	0.49	0.50	0.00	1.00
Political affiliation	99	0.81	0.40	0.00	1.00
Religion	306	0.26	0.44	0.00	1.00
Ethnicity	300	0.32	0.47	0.00	1.00
Social acceptance	282	0.67	0.20	0.00	1.00
Past recognition	282	0.94	0.15	0.00	1.00
Ongoing recognition	280	0.81	0.23	0.00	1.00
Justice	259	0.88	0.15	0.25	1.00
Autonomy	271	0.81	0.24	0.00	1.00
Scenario	302	0.50	0.50	0.00	1.00

*Note:* Not all variables will sum to 313 due to missing data. Gender (0 = W, 1 = M); Province (0 = SK, 1 = QC); Political affiliation (0 = right, 1 = left); Religion (0 = no, 1 = yes); Ethnicity (0 = no, 1 = yes); Social acceptance (0 = low acceptance, 1 = high acceptance); Past recognition (0 = low, 1 = high); Ongoing recognition (0 = low, 1 = high); Autonomy (0 = low, 1 = high); Justice (0 = low, 1 = high).

The majority of respondents in the study identified as women (78.5%), and 21.5% identified as men. The average income is $65,692.45 CAD^6^ (SD = 31,605.53). Approximately half of the respondents lived in Quebec (49.1%), while the other half resided in Saskatchewan (50.9%). Most of the sample did not consider themselves to belong to a particular ethnicity other than Canadian. Among those who indicated their ethnicity, most (77%) are of European origin. Thus, we can assume most of the sample was White, which reflects our selection criteria. The variable of ethnicity was excluded for further analysis given the lack of variance in the sample. A significant proportion (74%) reported that they do not have any religious affiliation. However, among those who did indicate their religious affiliation (*n* = 76), the majority (82%) identified as Christians.

The majority of respondents (94%) acknowledges past victimization, while a lower percentage (81%) recognizes ongoing victimization. This difference in recognition between past and ongoing victimization is statistically significant (*t* = 8.153, *p* < 0.001). The majority of respondents support both Autonomy (81%) and Justice measures (88%). However, there is a significant difference in support between the two, with Autonomy receiving less support compared to Justice (*t* = 4.483, *p* < 0.001).

The first section of the survey includes sociodemographic questions. These questions cover various aspects such as age, gender (coded as 0 for women and 1 for men), income (coded using the middle point value of each category), and province of residence (coded as 0 for Saskatchewan and 1 for Quebec). Respondents were asked if they identify with a political party, religion, and ethnicity (coded as 0 for ‘No’ and 1 for ‘Yes’). If respondents indicated ‘Yes’, they were requested to specify their answer. Political affiliation (*n* = 99) is coded as 0 for right and 1 for left. Due to the high skewness observed in the models involving political affiliation, we decided to exclude this variable from further analysis. Only 99 out of 313 respondents answered this question, and among those who did, a majority (81%) identified as leaning left on the political spectrum, likely due to our sample being composed of students. Including this variable in the analysis would introduce bias. All variables are coded on a scale of 0 to 1 to facilitate the subsequent analysis.

After the sociodemographic questions, participants’ perceptions toward reconciliation were assessed on Likert-type-scale questions (ranging from 1 for strongly agree to 5 for strongly disagree). All participants in the study were presented with a statement of apology to Indigenous Peoples made by the Canadian Prime Minister. Besides recognizing historical victimization, the purpose of this statement was to provide all respondents with factual information about the residential school system, ensuring that they had a common understanding of its historical context for the subsequent questions in the survey. Following that, participants were divided into two groups. One group was presented with a fictitious statement from a fictitious Indigenous leader expressing thanks for the apology and that it is a positive step forward (Scenario 1):

I am filled with hope that this action by the Canadian government, and the magnitude of the words it has chosen to transmit this apology will mean that this sad part of our collective and national history comes to an end. This apology reflects the desire for progress and healing. I congratulate the Prime Minister in particular because this the first time that the Canadian government officially apologized for all of the violence that occurred in the context of the Indian Residential Schools, whether it be sexual, physical or psychological. This statement by the PM, and in the name of Canada, is just, moral and profoundly humane. (Joseph Clermont, Inuit Leader)

The other group was presented with a fictitious statement that not only acknowledges the apology but also conveys that Canadians are inherently moral and good people (Scenario 2), thereby tapping into social acceptance:

I am filled with hope that this action by the Canadian government, and the magnitude of the words it has chosen to transmit this apology will mean that this sad part of our collective and national history comes to an end. This apology reflects the desire for progress and healing. I congratulate the Prime Minister in particular because this the first time that the Canadian government officially apologized for all of the violence that occurred in the context of the Indian Residential Schools, whether it be sexual, physical or psychological. This statement by the PM, and in the name of Canada, is just, moral and profoundly humane. *It reflects the goodness of Canadians and Canadian values, such as fairness, equity, and democracy.* (Joseph Clermont, Inuit Leader)

After the scenarios, participants were presented with a series of closed-ended Likert-type- scale questions (ranging from 1 for strongly agree to 5 for strongly disagree). These questions aim to evaluate participants’ perceptions of social acceptance, recognition of past and ongoing victimization, and support for self-determination and justice measures. The survey also includes open-ended questions at the end.

### Social acceptance

Based on the work of Shnabel et al. (2009), the measurement of social acceptance is captured through four items after being exposed to both statements from the Indigenous leader. These items include (a) Acceptance of non-Indigenous Canadians, (b) Understanding of non-Indigenous Canadians’ emotions, (c) Recognition of non-Indigenous Canadians as human beings just like us, and (d) Empathy toward non-Indigenous Canadians. Participants are asked to indicate their level of agreement with each statement on a five-point scale, ranging from ‘No, not at all’ to ‘Yes, very much’. Cronbach’s alpha for this scale is 0.76, which indicates acceptable construct validity. This coefficient suggests a satisfactory level of internal consistency and reliability for the scale (Cronbach and Meehl, 1955; Tabachnick and Fidell 2019).

## Outcome variables

### Recognition of ongoing victimization

Based on the work by Twali et al. (2020), we developed a scale to assess the recognition of ongoing victimization. The scale consists of three items: (a) Indigenous peoples experience psychological pain because of the Indian Residential School policy, (b) Indigenous peoples experience physical pain because of the Indian Residential School policy and (c) Indigenous peoples experience economic pain because of the Indian Residential School policy. Participants were asked to rate their agreement with each statement on a five-point scale, ranging from strongly disagree to strongly agree. The responses were then coded on a continuous scale from 0 to 1, with 0 indicating low recognition and 1 indicating high recognition. Cronbach’s alpha for this scale is 0.87, which indicates good construct validity.

### Recognition of past victimization

Recognition of past victimization is a scale consisting of three items: (a) Indigenous peoples experienced psychological pain, (b) Indigenous peoples experienced economic pain, and (c) Indigenous peoples experienced physical pain. Participants are asked to rate their agreement with each statement on a five-point scale, ranging from strongly disagree to strongly agree. The responses were then coded on a continuous scale from 0 to 1, with 0 indicating low recognition and 1 indicating high recognition. Cronbach’s alpha for this scale is 0.90, which indicates good construct validity.

### Support for justice

Support for justice is measured using a scale inspired by transitional (Quinn, 2009) and transformative justice theory (Hoddy and Gready, 2020). The scale comprises four items that assess the concept of justice following the Indian Residential Schools (IRS): (a) Knowing the truth about what happened, (b) Establishing a historical record of what happened, (c) Rehabilitation of the victims, and (d) Implementing social justice reforms such as economic resource redistribution. Participants rated their level of agreement with each statement on a five-point scale, ranging from strongly disagree to strongly agree. The responses are then transformed into a continuous scale ranging from 0 to 1, where 0 indicates high support for justice and 1 indicates high support for justice. The scale demonstrates acceptable construct validity with a Cronbach’s alpha coefficient of 0.78.

### Support for autonomy

Support for autonomy is assessed using a scale tapping in self-determination rights (UN, 2007). The scale consists of two items: (a) Indigenous Peoples should not have control over the education of their youth (reverse-coded) and (b) Indigenous Peoples should not have control over their land and resources (reverse-coded). Participants are asked to rate their agreement with each statement on a five-point scale, ranging from strongly disagree to strongly agree. The responses were then coded on a continuous scale from 0 to 1, with 0 indicating low support for autonomy and 1 indicating high support for autonomy. The scale demonstrated good internal consistency, with a Cronbach’s alpha coefficient of 0.80.

## Analysis

A *t*-test was performed to examine whether there were significant differences between the messages conveyed by Scenario 1 and Scenario 2. *t*-tests indicate that there is a significant difference in social acceptance between Scenario 1 and Scenario 2. In Scenario 1, the estimated mean for Social acceptance is 0.649, with a *t*-statistic of 40.3 (*p* < 0.000), suggesting evidence against the null hypothesis. The confidence interval (0.617 to 0.681) indicates that the true mean for social acceptance in Scenario 1 is likely to be within this range. Similarly, in Scenario 2, the estimated mean for social acceptance is 0.698, with a *t*-statistic of 39.5 (*p* < 0.000). The confidence interval (0.663 to 0.733) suggests that the true mean for social acceptance in Scenario 2 is likely to be within this range. These results indicate that both scenarios have significantly different levels of social acceptance, supporting the notion that there are variations in social acceptance between the two scenarios.

Although there are differences observed between the scenarios, particularly with Scenario 2 appearing to have conveyed slightly better social acceptance as it was intended, it is important to note that these differences are relatively small and likely underpowered, meaning the sample size may not be sufficient to detect significant differences accurately^7^. It is important to approach the interpretation of the results with caution, as the observed relationships in the analysis may be underpowered due to the small sample size (Arel-Bundock et al., 2022). This limitation could potentially lead to an overestimation or underestimation of the true relationship between the variables. Furthermore, the level of social acceptance conveyed in both scenarios is low. There could be several reasons for this observation. One possibility is that the statements used in the assessment are too similar, leading to a lack of distinctiveness in the responses. Both statements were likely to have conveyed the same level of acceptance to the respondents. Another possible explanation is that the scenarios may not have effectively conveyed social acceptance, but instead emphasized a sense of hope and new beginnings.

We calculated the correlations (see Table 2) to further investigate the relationships among the independent variable, outcomes, and the scenarios. The correlations between the scenarios and the variables, namely, Past recognition, Ongoing recognition, Autonomy, and Justice, were found to be low. This suggests that there is limited association between the scenarios and these variables. A correlation coefficient of 0.12 indicates a weak positive relationship between the scenarios and social acceptance. However, the strength of the correlation is considered to be weak, suggesting that the scenarios have only a limited influence on social acceptance.

**Table 2.** Correlations.

	Social acceptance	Past recognition	Ongoing recognition	Justice	Autonomy	Scenario
Social	1	.	.	.	.	.
acceptance						
Past	0.12	1	.	.	.	.
recognition						
Ongoing	0.17	0.44	1	.	.	.
recognition Justice	0.34	0.48	0.55	1	.	.
Autonomy	0.09	0.33	0.32	0.27	1	.
Scenario	0.12	*−*0.08	0.02	0.03	0.03	1

Social acceptance shows a positive correlation with the recognition of past and ongoing victimization as well as support for justice measures, but it exhibits a weaker correlation with autonomy. In addition, recognition of past and present victimization is correlated with both Justice and Autonomy, although the correlation is somewhat weaker for Autonomy, as indicated in Table 2. This suggests that recognition may play a significant role in influencing attitudes toward Autonomy and Justice measures. For this analysis, we will concentrate on the variable ‘Social acceptance’ as our primary focus. It exhibits positive correlations with the outcome variables, particularly Ongoing recognition and Justice, which demonstrate stronger associations. Our goal is to better understand the relationship between Social acceptance and outcome variables.

We proceeded by estimating eight regression models, each focusing on a different outcome variable. All regression analyses were conducted using linear regression with robust standard errors in R, which accounts for potential violations of assumptions and provides more reliable estimates. For each outcome variable, two models were constructed. In the first model, the relationship between the outcome variable and Social acceptance is examined. In the second model, control variables were included to address potential confounding factors. The estimated coefficients and relevant statistics are summarized in Table 3.

**Table 3.** OLS models.

	Past victim		Ongoing victim		Justice		Autonomy	
	(1)	(2)	(3)	(4)	(5)	(6)	(7)	(8)
Social acceptance	0.088*	0.067	0.189*	0.244**	0.240***	0.231***	0.105	0.175*
	(0.041)	(0.044)	(0.074)	(0.086)	(0.057)	(0.059)	(0.075)	(0.088)
Num. Obs.	278	219	276	217	256	203	267	211
R2	0.015	0.057	0.028	0.115	0.112	0.192	0.008	0.090
Controls	No	Yes	No	Yes	No	Yes	No	Yes

*Note*: Robust standard errors are in parentheses.

Control variables in models 2, 4, 6 and 8 include Age, Gender, Income, Province, and Religion.

*p* *<* 0.1, *p *<* 0.05, **p *<* 0.01, ***p *<* 0.001. Asterisks indicate levels of statistical significance.

Based on the predicted correlations of Table 2, the linear regression analyses also suggest positive relationships between Social acceptance and the outcome variables. However, the R-squared values indicate that only a small proportion of the variance is explained by the predictors in the models. Models 2, 4, 6, and 8, which incorporate control variables, provide more precise estimates with smaller standard errors. These models take into account potential confounding factors and provide a more accurate understanding of the relationship between Social acceptance and the outcome variables.

A significant relationship is observed between social acceptance and past victimization, although this relationship does not remain significant when controlling for other variables in Model 2. A significant relationship is observed between Social acceptance and Ongoing recognition of victimization (0.244, *p* < 0.01, SD = 0.086). The most significant relationship observed is between Social acceptance and Justice, with a coefficient of 0.240 (*p* < 0.001, SD = 0.057). Support for Autonomy is weakly associated with Social acceptance, as evidenced by the non-significant relationship in Model 7. However, in Model 8, when control variables are included, a significant relationship emerges between Autonomy and Social acceptance. The inclusion of control variables helps to account for the significant results observed in the analysis.

**Figure 1.**
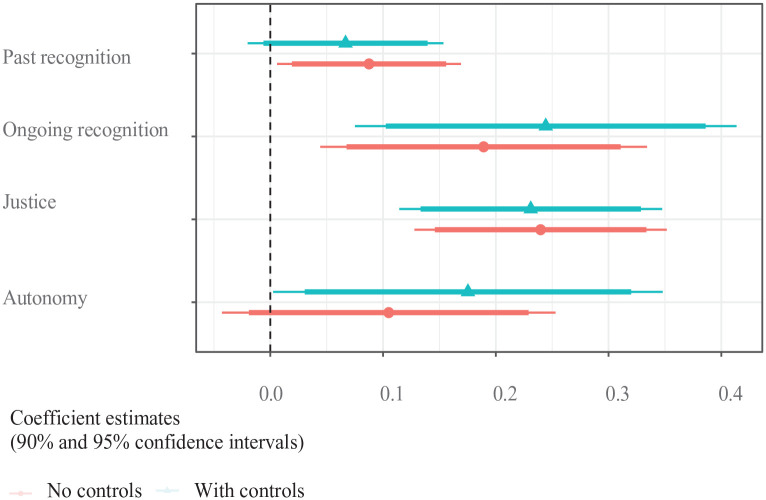
Social Acceptance Coefficient estimates.

Figure 1 displays the coefficient estimates along with confidence intervals for both the models with control variables and the models without control variables. These coefficient estimates are a graphic representation of the data presented in Table 3. This figure demonstrates a positive correlation between Social acceptance and the outcome variables at 90% and 95% confidence intervals, meaning the actual parameter value is likely to fall somewhere within that extensive range among our sample. The relationships between Social acceptance and Ongoing recognition and Justice appear to be more precise. Wider intervals signify greater uncertainty and are symptomatic of a smaller sample size.

## Discussion

The influence of social acceptance on the recognition of past victimization seems to be less significant. This could be attributed to the fact that a majority of the respondents may have already achieved the initial level of recognition (Twali et al., 2020). As indicated in Table 1, the majority of respondents acknowledged past victimization. Recognition of past victimization may pose less of a threat to one’s identity and is more widely acknowledged in Canada, which is also consistent with research (Environics Institute Survey Research, 2021; Peetz et al., 2010; Wohl et al., 2019). Respondents may have already been sensitized and educated due to historical events preceding the survey. Between January and March 2020, a series of civil disobedience demonstrations took place in Canada to oppose the construction of the Coastal GasLink Pipeline (CGL) across 190 kilometers of unceded Wet’suwet’en First Nation territory in British Columbia. These protests received extensive media coverage and touched broader issues such as Indigenous land rights, police conduct, land conservation, and the environmental consequences of energy projects (Gobby et al., 2022). In addition, the choice of a student sample presents another key consideration. As previously mentioned, students are in a transitional phase of life, where both their identities and material circumstances are less settled compared to later stages. This makes them more susceptible to change, potentially more liberal, more open to influence, and less materially invested in issues related to Indigenous victimization and redress. This context is crucial to consider, as it may amplify the effects of social approval while reducing variability in their recognition of injustice and support for remedial measures. Future research should take this into account when interpreting findings.

It is important to note that we did not directly compare the perceptions of respondents from Quebec and Saskatchewan on Indigenous issues, as the small sample size could introduce bias into the analysis. The selection of these two provinces aimed to capture a diverse range of perspectives, given that previous research has highlighted differences in views on Indigenous issues based on provincial identities (Beauvais, 2022; Environics Institute Survey Research, 2019; Letourneau, 2024). This remains an important consideration for future research exploring perspectives shaped by provincial identities.

Table 3 and Figure 1 provide evidence of a positive relationship between social acceptance and the recognition of ongoing victimization. This finding sheds light on the second level of acknowledgment and how it can be encouraged (Twali et al., 2020). While the majority of participants in the study recognized the presence of ongoing victimization, their level of acknowledgment was lower compared to past victimization, which concurs with past surveys among Canadians (Environics Institute Survey Research, 2021). In this regard, social acceptance may play a role in facilitating the recognition of ongoing victimization. When acknowledging ongoing victimization poses a threat to the advantaged group identity (Shnabel et al., 2021; Wohl et al., 2019), social acceptance by the disadvantaged group can help mitigate that threat. Social acceptance may play a significant role in the recognition of ongoing victimization.

Social acceptance may also play a role in cultivating a sense of responsibility toward addressing persistent inequalities by supporting justice measures, aligning with the third level of recognition (Twali et al., 2020). To support reparative measures, the perpetrator must recognize inequality and be willing to take responsibility for the harm caused (Iqbal and Bilali, 2018; Twali et al., 2020). This also seems to be suggested by our findings. Although we did not measure personal responsibility directly in the survey, prior to the scenarios, the message from the prime minister described in detail how the residential school system affected and still affects Indigenous peoples to this day, as well as the responsibility of the state and Canadians in it. We could thus argue that respondents who recognized past and ongoing victimization and also supported justice and autonomy measures felt a sense of responsibility in rectifying this situation. Further research is needed to explore the link between social acceptance, responsibility, and support for reparations.

Our study did not measure the fourth level of acknowledgment, ‘Negative Identity Internalization’, due to its complexity and difficulty to capture, but it is plausible to hypothesize that Canada may not have fully reached this level of acknowledgment at present. At this level, society as a whole must recognize its responsibility for past and ongoing inequalities as an integral part of the Canadian identity. Maintaining a safe distance from the past is a defensive mechanism that can hinder many individuals from engaging in this process (Peetz et al., 2010). While social acceptance may contribute to increasing empathy in individual interactions in the short term, negative identity internalization is a process that requires time, collective effort, and governmental acknowledgment. At the most basic level, it would require a reform of the education system to provide Canadians with an accurate understanding of history (Quinn, 2009). Currently, systemic and structural racism, as well as genocide, remain contentious topics in Canada, which indicates the fragility of the state of recognition and underscores that there is still a considerable journey ahead of us.

Social acceptance is associated with favorable attitudes toward justice following the IRS. The majority of respondents expressed support for various measures, including understanding the truth, establishing a historical record, rehabilitating victims, and implementing social justice reforms like redistributing economic resources. These measures encompass a broad range of reparative actions that have already been implemented in Canada, indicating a potentially high level of social acceptability for them. It is worth noting that respondents may interpret the items differently. For example, the concept of victim rehabilitation may be understood as financial compensation by some, while others may perceive it as psychosocial support services. Most of those items could be considered weaker justice measures (Lincez, 2022; Nagy, 2013). However, the fourth item is in line with transformative justice theory (Nagy, 2022), emphasizing the importance of addressing systemic inequalities through social justice reforms. Nonetheless, it is promising to observe that a majority of respondents (see Table 1) are in agreement with these measures and that social acceptance may play a role in encouraging support toward justice measures. The strong positive correlation between recognition of ongoing victimization and justice suggests that perhaps it is because one recognizes ongoing victimization that justice is more relevant for this group.

Feeling socially accepted by Indigenous Peoples may be related to a tendency to support mostly weaker Justice measures, but it does not necessarily indicate a willingness to support Autonomy. Autonomy should be considered as a crucial Indigenous right (UN, 2007), but received less support from respondents compared to Justice. Social acceptance is associated with favorable attitudes toward Autonomy only when control variables are added, suggesting it may play less of a role in promoting Autonomy. Insufficient support for Autonomy is evident in the qualitative analysis of the survey. When participants were asked about potential solutions to improve the economic situation of Indigenous Peoples, the majority of respondents expressed a preference for a collaborative approach with the government compared to self-determination. A key takeaway from this study is that while social approval facilitates narrower, less institutionally disruptive forms of recognition and policy, it falls short of challenging broader conceptions of settler-Indigenous relations—particularly the power dynamics tied to autonomy. Further research is needed to establish a clear understanding of the relationship between Social acceptance and Autonomy. Autonomy may be considered as a drastic measure by some, and may elicit more defensiveness. Tying it to Twali’s fourth level of recognition of victimization, fully supporting this measure entails taking responsibility and internalizing past and historical injustices as an integral part of one’s identity (Twali et al., 2020). Achieving this may prove challenging, even in the presence of social acceptance from Indigenous Peoples, echoing Chris Hiller’s work (2016) on spirals of unsettlement, where acknowledgment is often met with resistance, and settlers may experience cumulative shifts in consciousness that push them into action or lead them to reflect inwardly on their complicity in historical injustices, potentially reinforcing defensiveness and support for the status quo.

The findings support partially the needs-based model. To encourage reconciliation, it is important to include social acceptance of non-Indigenous Canadians. Social acceptance can serve as a mechanism to mitigate identity threats in the short term and foster recognition of collective victimization and support for justice measures (Shnabel et al., 2021). However, in the long term, it is important to address negative identity internalization that may hinder lasting progress and transformative change. The scenarios conveyed acceptance, empathy and understanding to non-Indigenous Canadians regarding past atrocities. It is important to recognize that the process of reconciliation is not always that harmonious and Indigenous Peoples may not necessarily socially accept or forgive non-Indigenous Canadians. Reconciliation should rather ‘unsettle’ and challenge Canadians to confront uncomfortable truths. It is a process that necessitates personal introspection and active engagement from Canadians themselves rather than external motivation. Nevertheless, social acceptance may help take this journey a significant step forward.

## Conclusion

This paper examined the influence of social acceptance by Indigenous Peoples on the willingness of non-Indigenous Canadians to recognize ongoing victimization and acknowledge the autonomy of Indigenous Peoples. Data from a survey of 313 participants were analyzed to explore the relationship between social acceptance and (a) recognition of victimization, (b) support justice measures, and (c) autonomy. The results indicated a positive correlation between social acceptance and both recognition of victimization and support for justice measures. However, no significant association was found between social acceptance and support for autonomy. Feeling socially accepted by Indigenous Peoples may be related to a tendency to support weaker justice measures, but it does not necessarily indicate a willingness to support autonomy. Further research should explore the relationship between social acceptance and support for justice measures and autonomy.

These findings support partially the needs-based model, suggesting that social acceptance plays a role in promoting reconciliation. It serves as a mechanism to alleviate identity threats in the short term and facilitates recognition of collective victimization and support for justice measures, enabling less institutionally disruptive forms of recognition and policy. However, it falls short of challenging broader understandings of settler-Indigenous relations, particularly the power dynamics linked to autonomy. There remains a significant gap between supporting narrow measures of redress and engaging with the larger framework of Indigenous autonomy and land restitution. It is important to recognize that reconciliation is a multifaceted and intricate process that requires more than just social acceptance. It should go beyond comfort and instead challenge Canadians to confront uncomfortable truths, fostering a deeper understanding and commitment to transformative change.

Despite this complexity, social acceptance can be an important initial step on the journey toward reconciliation. Highlighting the moral compass and openness of Canadians could serve as a catalyst for discussions on Indigenous issues and reparations. It is important that the responsibility of social acceptance is not solely shouldered by Indigenous communities; the government should also take a leading role. By appealing to the so-called goodness of Canadians (Henry et al., 2010; Regan, 2010), despite historical wrongs, the government could potentially drive educational initiatives to educate them about colonialism, its lasting impacts, and their part in the reconciliation process. Additional research is necessary to explore the role of the government in the reconciliation process.

It is crucial to take into account the impact of underpowered analyses on the findings. It is important to interpret the results with caution, as the observed relationships may be influenced by the small sample size and could potentially lead to an over-estimation or underestimation of the true relationship between the variables. Another limitation is that the sample was composed exclusively of students. It is recommended to conduct this research among a representative sample of Canadians based on power analysis. A larger and more diverse sample would provide a more comprehensive understanding of the perspectives and opinions of Canadians regarding reconciliation. Surveys inherently have limitations, including potential issues such as social desirability bias and limited attention spans. To mitigate these limitations, it is crucial to ensure that the scenarios used in the survey convey clear and distinct messages.

## Notes

1. The Indian Residential School system is the most documented and widely recognized manifestation of colonial assimilation in Canada (TRC, 2015), but it is part of a broader continuum of ongoing colonial structures and practices.

2. It is important to acknowledge the existence of other significant reports, such as the Royal Commission on Indigenous Peoples, which began its work in the 1990s following the Oka Crisis. While our focus is primarily on more recent reports, it is crucial to recognize the contributions of earlier inquiries.

3. See https://ici.radio-canada.ca/nouvelle/1711207/canada-racisme-systemique-trudeau-grc-lucki-police

4. Article 4 of the (UN, 2007): Indigenous Peoples, in exercising their right to self-determination, have the right to autonomy or self-government in matters relating to their internal and local affairs, as well as ways and means for financing their autonomous functions. Bill C-15 implemented the UNDRIP in Canada in 2021.

5. Disadvantaged and advantaged group are terms that apply to individuals who perceive themselves as being affiliated with an advantaged or disadvantaged group (Shnabel et al., 2021).

6. $1 CAD is worth approximately US$0.69.

7. Due to the small sample size, it was not possible to conduct a comparative analysis between Quebec and Saskatchewan. Grouping the data could introduce potential bias in the results, so it is preferable to analyze the data as a whole to minimize any potential biases (Arel-Bundock, 2020: 160).
